# Training and Transfer of Training in Rapid Visual Search for Camouflaged Targets

**DOI:** 10.1371/journal.pone.0083885

**Published:** 2013-12-27

**Authors:** Mark B. Neider, Cher Wee Ang, Michelle W. Voss, Ronald Carbonari, Arthur F. Kramer

**Affiliations:** 1 Department of Psychology, University of Central Florida, Orlando, Florida, United States of America; 2 Beckman Institute for Advanced Science and Technology, University of Illinois Urbana-Champaign, Urbana, Illinois, United States of America; 3 Department of Psychology, University of Iowa, Iowa City, Iowa, United States of America; Centre de Neuroscience Cognitive, France

## Abstract

Previous examinations of search under camouflage conditions have reported that performance improves with training and that training can engender near perfect transfer to similar, but novel camouflage-type displays [1]. What remains unclear, however, are the cognitive mechanisms underlying these training improvements and transfer benefits. On the one hand, improvements and transfer benefits might be associated with higher-level overt strategy shifts, such as through the restriction of eye movements to target-likely (background) display regions. On the other hand, improvements and benefits might be related to the tuning of lower-level perceptual processes, such as figure-ground segregation. To decouple these competing possibilities we had one group of participants train on camouflage search displays and a control group train on non-camouflage displays. Critically, search displays were rapidly presented, precluding eye movements. Before and following training, all participants completed transfer sessions in which they searched novel displays. We found that search performance on camouflage displays improved with training. Furthermore, participants who trained on camouflage displays suffered no performance costs when searching novel displays following training. Our findings suggest that training to break camouflage is related to the tuning of perceptual mechanisms and not strategic shifts in overt attention.

## Introduction

Visual search often feels effortless. However, we sometimes realize the constraints imposed on our search abilities when we engage in difficult search tasks, such as when trying to find our missing keys on a messy tabletop, or the baby’s pacifier dropped somewhere amongst his or her scattered toys. These types of difficult search tasks are also encountered in more pressing contexts. For example, a medical doctor must search an ultrasound image and determine if a tumor is lurking in the midst of other benign fibrous tissue and drivers must be constantly vigilant for undefined, but rapidly emerging hazards. All of these examples share important common threads. The target being searched for is similar to the distracter items amongst which it is arrayed, the background upon which it is presented, or both. In all cases, search is being conducted for a target signal in correlated noise of varying degrees – in the most challenging examples the signal and noise distribution may be nearly overlapping. In many of the contexts in which these types of search tasks occur, rapid and/or accurate execution is critical. Furthermore, the possibility of improving performance in such tasks without the need for extensive long-term experience (e.g., radiologists require years of on the job practice before becoming experts in their craft) could provide tremendous real world value. This begs the question: can we improve performance with training on search tasks where the target is concealed and particularly in cases where it shares common features with the background.

In recent years, a number of studies have considered the problem of target-background similarity in search. In a series of studies characterizing the effect of background on search processes, Wolfe and colleagues found that increasing target-background similarity affected the recognition processes responsible for determining whether a given collection of features was the target object or not [2]. Specifically, they suggested that information about objects accrues at a slower rate when the target and background share common features, causing the decision threshold to be reached more slowly. In another study in which eye movements were recorded, Neider and Zelinsky experimented with varying the target-background similarity to create search arrays in which the target was camouflaged to lesser or greater degrees [3]. They found that observers made more fixations to the background as target-background similarity increased, suggesting that observers might mistake portions of the background for the target when the target and background are similar. Additionally, Neider and Zelinsky noted that observers made an increasing proportion of their eye movements to salient distractor items as target-background similarity increased. This finding was particularly surprising given the nature of the search task (the target was always camouflaged to some degree); the target was never highly salient. As a result, an intuitive top-down strategy in the search task would have been to disregard any salient items, as they could only be distractors. Neider and Zelinsky interpreted the data pattern as evidence that attentional processes interact with object-based representations; in the case of a camouflaged target the target itself is not overtly object-like.

Although Wolfe and colleagues [2] and Neider and Zelinsky [3] provided baseline characterizations of how camouflage influences search processes, they did not examine whether observers could improve their ability to detect camouflaged targets. To fill this gap in the literature, Boot, Neider and Kramer explored whether training could improve camouflage target sensitivity and whether training participants to detect one set of camouflaged targets could engender transfer to a novel set of camouflaged targets ( [1]; also see [4], for a study of age-related differences in training and transfer of search for camouflaged targets). Specifically, they had one group of participants train on a camouflage visual search task (using a nearly identical paradigm to Neider and Zelinsky [3], and another group of participants train on a visual search task using the same objects, but arrayed on a homogenous background (no camouflage). After training both groups performed the camouflage versions of the search task, but with a novel set of targets, backgrounds, and distractors. Interestingly, prior to beginning their training, the camouflage search group was instructed that a good strategy for locating the target might be to ignore salient objects and focus on background regions of the display. Both groups showed performance improvements during their training; camouflage trained participants improved at searching for camouflage items and non-camouflage trained participants improved at searching for non-camouflaged items. Eye movement analyses showed that the camouflage training group did not preferentially restrict their eye movements to background regions, despite being instructed that doing so might improve their task performance. None the less, over training they were able to fixate the target with fewer eye movements and generated responses (i.e., a button press) more rapidly once they fixated the target. Perhaps even more importantly, participants in the camouflage training group showed excellent transfer to novel target objects; search performance with a novel set of objects was equivalent to that on the trained objects. The finding of near perfect transfer was particularly surprising given that transfer of training in perceptual tasks is often narrow and very specific [5]–[7]. In speculating upon the basis for their camouflage group’s training improvements and transfer benefits, Boot and colleagues noted that despite instruction that avoiding salient objects might be a good search strategy, in many cases participants still directed their eye movements towards salient objects. This suggests that training improvements were not the result of some overt top-down strategy shift. However, the authors could not rule out the possibility that covert attentional mechanisms might be operating on background regions of the display. It is also important to note that camouflage trained participants were faster to make a button-press response after fixating a target with training. This data pattern is supportive of Wolfe and colleague’s conclusion that target-background similarity affects the decision making stage (i.e., recognizing that an item is a target or distractor, as opposed to a detection stage during which a set of candidate features that *might* be a target are identified; see [2], for an in-depth discussion of this possibility) of processing. In the case of Boot and colleagues’ training manipulation, it is possible that observer’s perceptual processes became better attuned to camouflage conditions over time, allowing for less noisy representations of the search display to be passed on to recognition processes. One possible candidate process for this tuning could be figure-ground texture segregation, which has been shown to be both associated with camouflage detection [8] and malleable via training [9].

In the present study we directly tested whether training improvements in previously reported studies for camouflaged targets were related to changes in low-level perceptual processes or higher-level shifts in overt search strategy. To do so, we trained participants to search for camouflaged or non-camouflaged targets, but whereas previous studies used self-terminating search tasks [1], [4], we used a rapid presentation paradigm where participants only viewed the display for 150 ms before making a target presence response. Participants were also required to maintain central fixation at all times and this was ensured using an eye tracker. As a result of our timing parameters, participants were highly unlikely to make search-related eye movements (rare cases in which eye movements did occur were omitted from analysis). If previously observed training improvements were related to overt strategy shifts (e.g., saccading to background regions preferentially) then we would expect to find limited or no training improvements in our task, given that the presentation was too rapid for eye movements. Alternatively, if training improvements are primarily related to changes in perceptual processes (e.g., figure-ground segmentation) then we would expect to observe similar training improvements to those found in our previous studies using free viewing paradigms. Additionally, we also included a test of transfer in our study to confirm previous reports of training of camouflage search on one set of objects producing benefits during search for novel camouflaged items, in the present case during covert search.

## Methods

### Ethics Statement

The study was conducted in accordance with protocols reviewed and approved by the University of Central Florida and the University of Illinois at Urbana- Champaign Institutional Review Boards, respectively.

### Participants

Forty-eight students (age 18–29, M = 21.6, SD = 2.75; 20 males) at the University of Illinois participated in the experiment. Participants were randomly assigned to one of the two training groups (24 participants in each group; camo group mean age = 22, 13 female; non-camo group mean age = 21, 15 female). All participants demonstrated normal or corrected to-normal acuity and color vision, as assessed with a Snellen chart and Ishihara plates respectively. Participants provided written informed consent and were paid $56 for their participation in seven 40- to 60-min sessions that took place over seven separate days within a span of three weeks.

### Apparatus and Stimuli

Displays were presented on a 21-inch CRT monitor with a resolution of 800 by 600 pixels (40cm by 30cm). An Eyelink II eye tracking system (SR Research, Inc.) sampled the position of each participant’s left eye at a speed of 250 Hz. Participants viewed the monitor from a distance of approximately 62 cm (visual angle of approximately +/−17.9° horizontally, and +/−13.6° vertically). A chin rest stabilized the head position and kept viewing distance constant. A Microsoft video game controller was used to collect responses.

**Figure 1:**
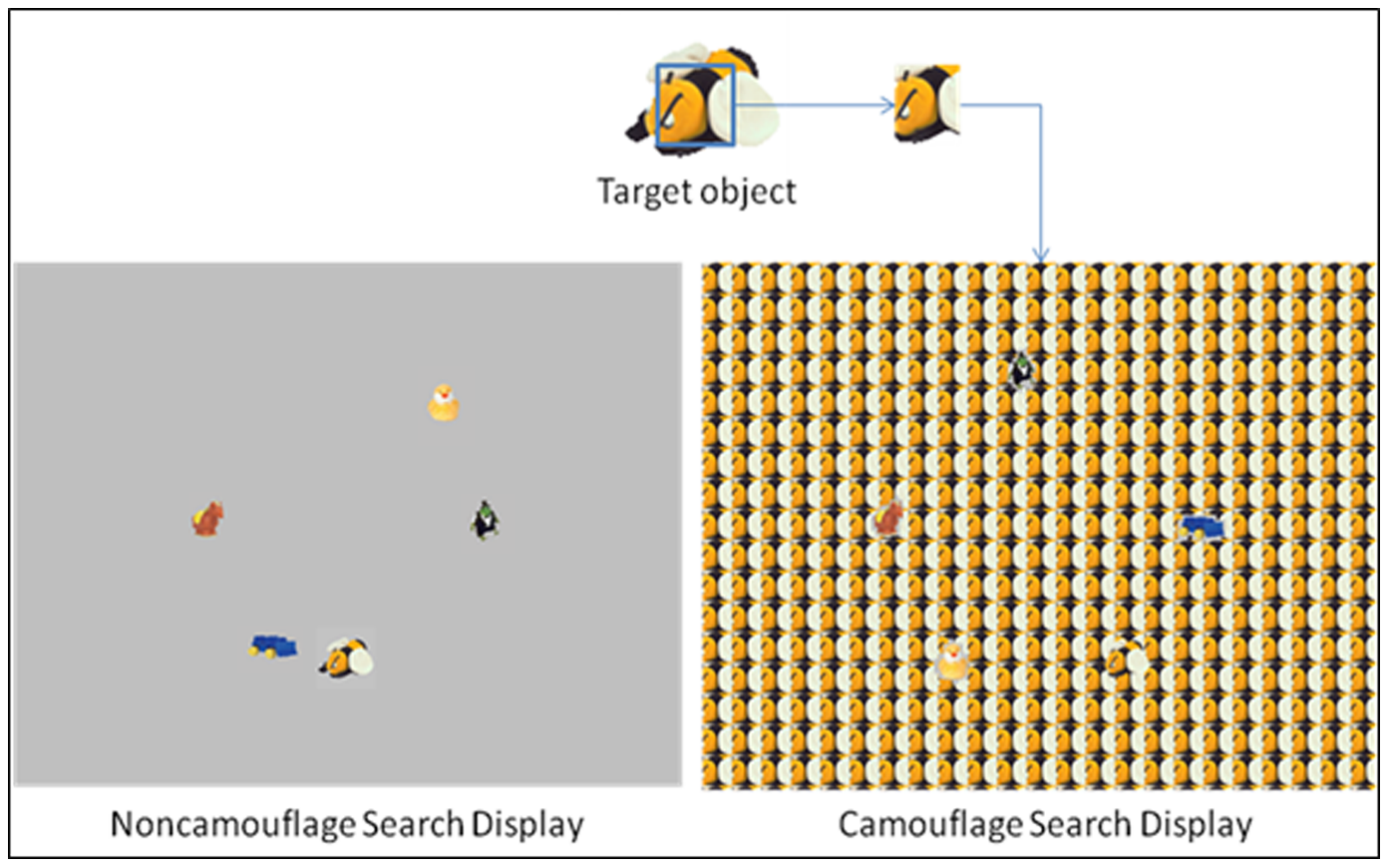
Sample search displays with both camouflage and non-camouflage backgrounds.

Stimuli were nearly identical to those used by Neider and Zelinsky [3]. Search items were selected from the Hemera Photo Objects database. Targets and distractors were 40 pictures of children’s toys, including dolls, stuffed animals, blocks, and toy vehicles. Each toy image was scaled to fit within an 80 by 80 pixel (visual angle of approximately 3.7° by 3.7°) bounding box (Figure 1). A corresponding camouflage background for each toy image was created by taking a 35 by 35 pixel square from the center of the toy image and tiling it across an 800 by 600 pixel background. In non-camouflage search displays, the toy images were superimposed on a homogenous gray background.

On each trial, the target image was randomly selected from one of two sets of objects. There were 20 objects in each Set A and B for use in either the pre- and post-training tests or training sessions. Distractors were randomly selected from the respective set, excluding the object that was selected as target in each particular trial. Targets and distractors were randomly assigned to any of 12 possible locations around the center of the screen, each at 150 pixels (6.9°) from the center of the screen.

### Design and Procedure

Each participant completed a pre-training test session, five training sessions and a post-training test session. At the pre-training test sessions, each participant completed two distinct search tasks; one on a non-camouflage background and the other on a camouflage background. Both search tasks used objects from either Set A or B and had 120 trials. Half of the participants in each training group began with a non-camouflage search display while the other half began with a camouflage search display. Similarly, half of the participants in each training group used Set A for the pre- and post-training tests and Set B for training, while the other half used Set B for the pre- and post-training tests and Set A for training. Following this initial evaluation, all participants completed five training sessions with 360 trials per session for a total of 1,800 training trials. As in previous studies [1], [4], half of the participants were trained with a non-camouflage background while the other half trained with a camouflage background. Following training, all participants again performed the same two search tasks as in the pre-training test session. For each participant, the presentation order of the search task (non-camouflage, camouflage) in pre- and post-training tests was reversed in order to mediate practice and sequence effects on test performance.

**Figure 2:**
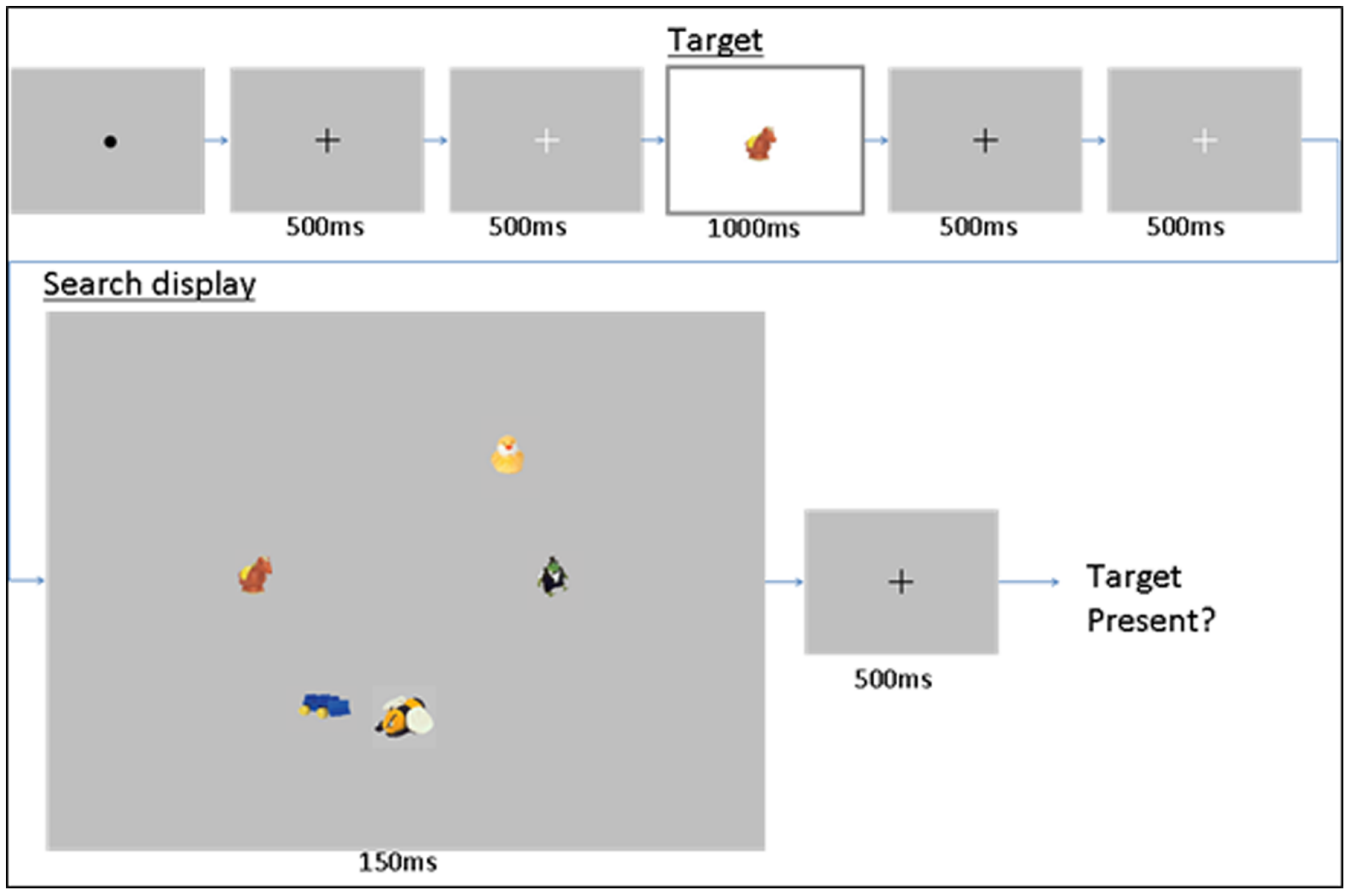
A typical trial sequence. Participants searched through either a non-camouflage (pictured) or a camouflage search display.

Participants began each search trial by fixing their gaze on a fixation dot at the center of the screen, and pressing any button on the game-pad to initiate that trial. The trial sequence would not begin unless the participant’s gaze was within 50 pixels from the center of the screen. Next, the participant received a sequence of displays related to the experimental trial (illustrated in Figure 2): (1) gray fixation cross (500 ms), (2) white fixation cross (500 ms), (3) target image (1000 ms), (4) gray fixation cross (500 ms), (5) white fixation cross (500 ms), (6) search display (150 ms), (7) gray fixation cross (500 ms) and (8) response screen. Multiple fixation crosses were presented throughout each trial sequence in order to ensure that the participant was ready for the onset of the search array, since the actual array itself was presented very briefly. On the final response screen in the sequence participants were reminded to pull the right trigger on the controller to respond that the target had been present in the search display and the left trigger if the target was absent. The search target was present on 50% of the trials. A trial was rejected if the participants’ gaze did not start the trial, end the trial, or remain within 50 pixels (visual angle of approximately 2.3°) from the center of the screen during the trial, or if they blinked during the time when the search display was on. Overall, ∼83% of trials were accepted and included in the analyses.

## Results

Analyses focused on the training sessions (pre-training compared to post-training) and then on the transfer effects (training session 5 vs. post-training test). In all cases, accuracy data were submitted to an omnibus ANOVA that included all factors, followed by more specific analyses focused on patterns in specific training groups where omnibus effects were significant. Given the tendency for accuracy data to violate the assumption of normality required for ANOVA, we also analyzed all accuracy data using an arc-sine transformation, which allows for a more normalized distribution. Importantly, all effects that reached significance when applied to untransformed data remained so when the same analyses were performed on arc-sine transformed data (all *p*’s <.05). As a result, we can be confident that our findings are associated with actual differences arising from our experimental manipulations rather than statistical anomalies arising from assumption violations. For the sake of clarity, all data and statistical analyses presented henceforth are reported in their original untransformed form. Additionally, in cases where ANOVA supports the null hypothesis, we also report p_BIC_(H_0_|D) [10]. This statistic provides an estimate of the posterior probability of the null hypothesis, allowing for conclusions to be drawn against or in support of the null hypothesis. A p_BIC_(H_0_|D) value greater than.5 supports the null hypothesis.

### Pre-Training Accuracy

**Figure 3:**
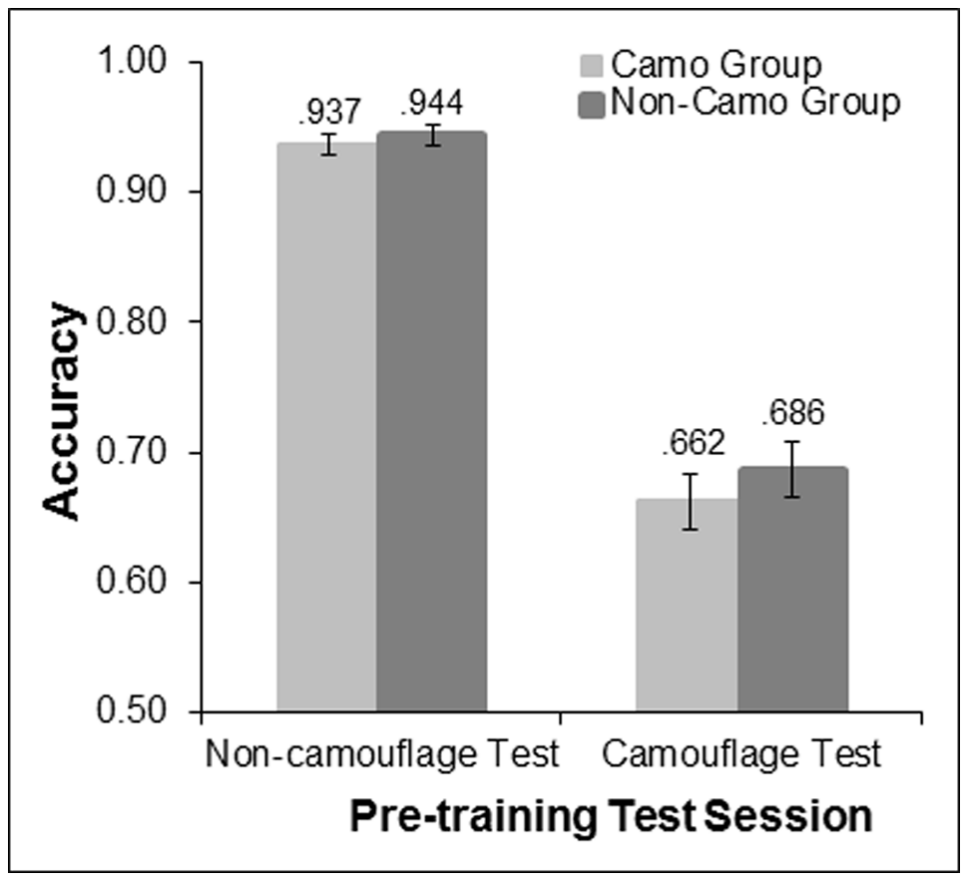
Mean accuracy for the camouflage and non-camouflage training groups in pre-training camouflage and non-camouflage background test sessions.

To examine whether the two training groups performance was equivalent at pre-test, the pre-training test accuracy data was entered into an ANOVA with type of test (non-camouflage vs. camouflage background), target presence (present vs. absent), and set size (3 vs. 5) as within-participants factors and training group (camo vs. non-camo) as a between-participants factor. As illustrated in Figure 3, accuracy did not differ significantly as a function of training group [*F* (1, 46) = .75, *p* = .39, p_BIC_(H_0_|D) = .82], indicating that both groups began their training at similar levels of search proficiency.

### Training Effects: Sessions 1 through 5

**Figure 4:**
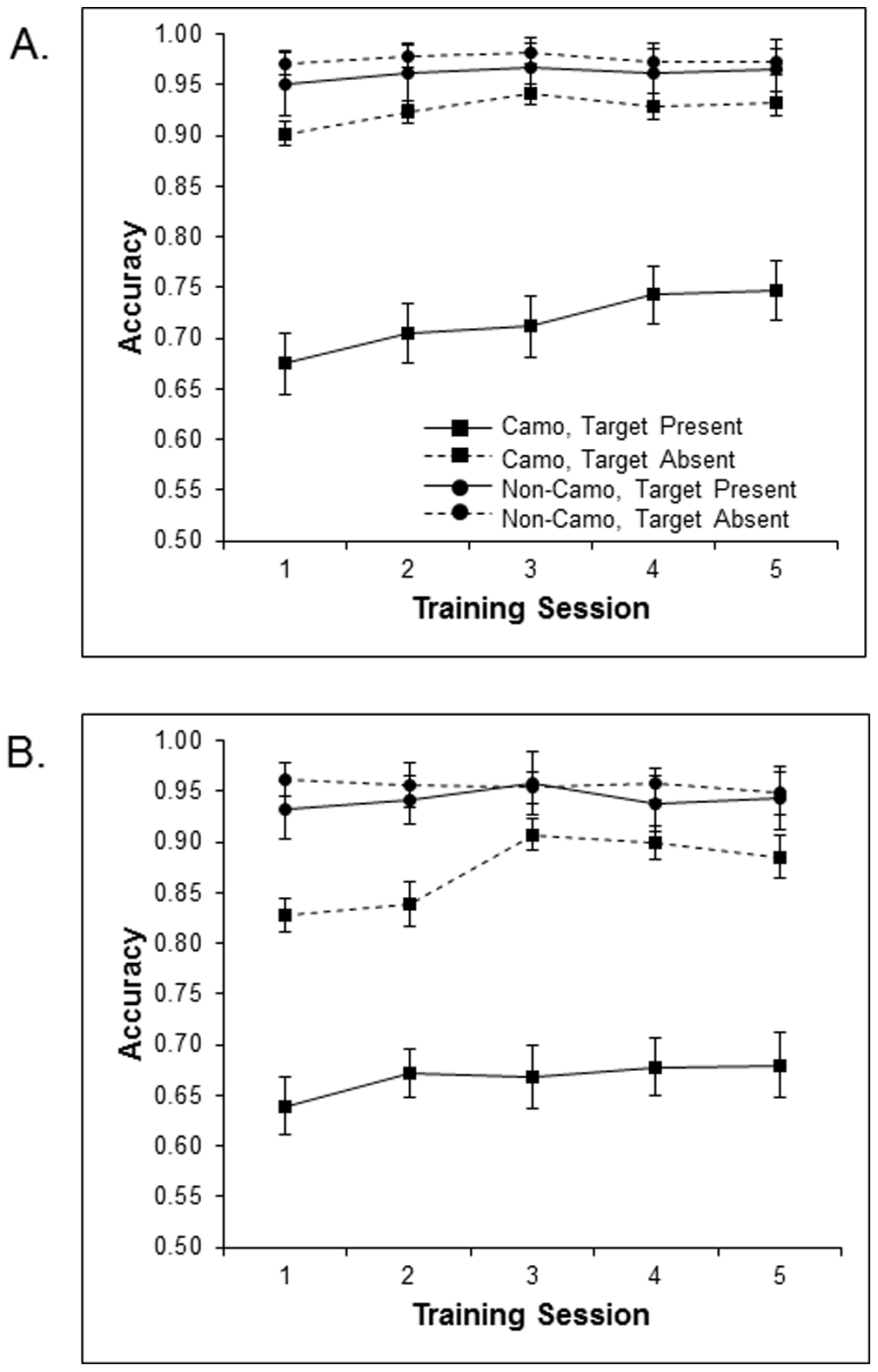
Mean accuracy across training sessions 1 through 5-camouflage training groups as a function of target presence at set sizes 3 (A) and 5 (B).

Accuracy data was entered into an ANOVA with training session (1, 2, 3, 4 and 5), target presence (present vs. absent), and set size (3 vs. 5) as within-participants factors and training group (camo vs. non-camo) as a between-participants factor. Figure 4 shows the mean accuracy of both groups over the five training sessions as a function of both target presence and set size. To account for a violation of the sphericity assumption (as indicated by Mauchly’s test, *χ* ^2^ (9) = 53.96, *p*<.001), data were analyzed using Greenhouse-Geisser (ε = .58) corrected degrees of freedom. We observed significant main effects of training session [*F* (2.34, 107.41) = 11.05, *p*<.001], target presence [*F* (1, 46) = 31.22, *p*<.001], set size [*F* (1, 46) = 146.00, *p*<.001], and training group [*F* (1, 46) = 50.80, *p*<.001]. Importantly, there was a significant interaction between training session and training group [*F* (4, 184) = 7.68, *p*<.001]; camouflage trained participants exhibited robust improvements in accuracy over training (∼5%), but non-camouflage trained participants did not (perhaps reflecting the non-camouflage training group’s near ceiling performance on non-camouflage search displays throughout training). The high accuracy observed for the no-camouflage training group throughout training is consistent with findings from previous studies using the same control condition [1], [4]. Both target presence [*F* (1, 46) = 23.90, *p*<.001] and set size [*F* (1, 46) = 30.50, *p*<.001] also interacted with training group. Finally, we found a significant interaction between training session, target presence and set size [*F* (4, 184) = 2.85, *p*<.05]. Broadly, our findings are consistent with those of Boot and colleagues [1].

### Transfer Effects: Pre- vs. Post-training Tests

To evaluate whether training improved search performance to stimuli beyond those that were trained on, accuracy data were entered into an ANOVA with test type (non-camouflage vs. camouflage background), target presence (present vs. absent), set size (3 vs. 5) and session (pre- vs. post-training) as within-participants factors, and training group (camouflage vs. non-camouflage) as the between-participants factor. Of central interest was the effect of type of training on the transfer test type. Main effects were observed for test type [*F* (1, 46) = 439.70, *p*<.001], target presence [*F* (1, 46) = 36.75, *p*<.001], set size [*F* (1, 46) = 31.59, *p*<.001] and session [*F* (1, 46) = 54.60, *p*<.001]. There was a significant interaction between test type, session, and training group [*F* (1, 46) = 4.67, *p*<.05], test type and target presence [*F* (1, 46) = 26.98, *p*<.001], and test type and session [*F* (1, 46) = 34.93, *p*<.001]. As test type interacted with multiple other factors, we analyzed the test types (non-camouflage transfer task and camouflage transfer task) separately to further interpret this three-way interaction.

#### Non-camouflage background test

**Figure 5:**
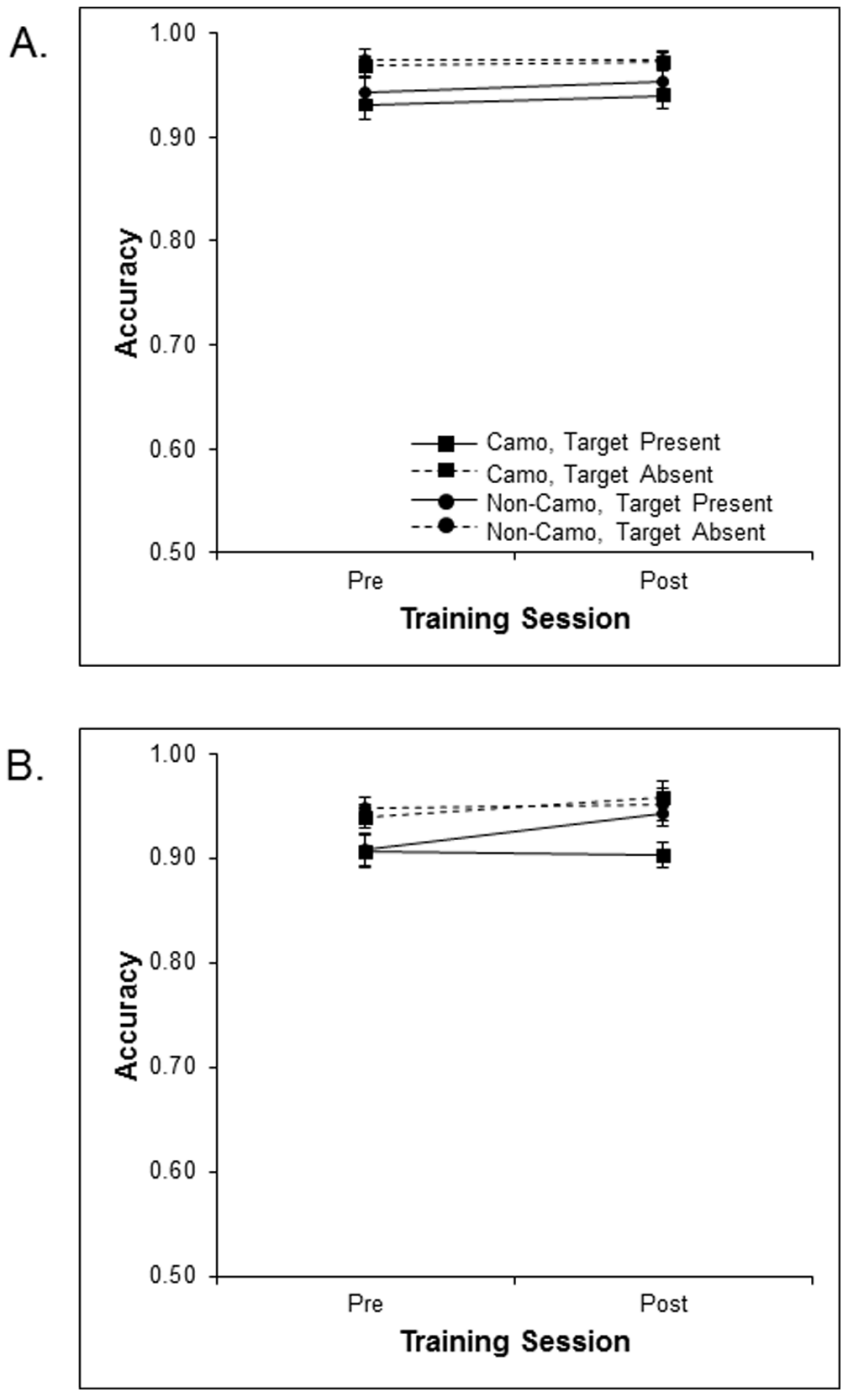
Mean accuracy across pre and post –training on untrained non-camouflage test displays for the camouflage and non-camouflage training groups as a function of target presence at set sizes 3 (A) and 5 (B).

Data from only the non-camouflage background test were entered into an ANOVA with target presence (present vs. absent), set size (3 vs. 5) and session (pre- vs. post-training) as within-participants factors, and training group (camouflage vs. non-camouflage) as a between-participant factor. Main effects of target presence [*F* (1, 46) = 16.10, *p*<.001] and set size [*F* (1, 46) = 32.97, *p*<.001] were observed. The lack of session [*F* (1, 46) = 2.50, *p* = .12, p_BIC_(H_0_|D) = .67] and training group [*F* (1, 46) = .83, *p* = .37, p_BIC_(H_0_|D) = .82] main effects suggest that neither training groups improved on the non-camouflage background (Figure 5). It is worth noting that both groups displayed high accuracies when searching non-camouflaged displays, suggesting that the absence of training improvement could reflect a ceiling effect.

#### Camouflage background test

**Figure 6:**
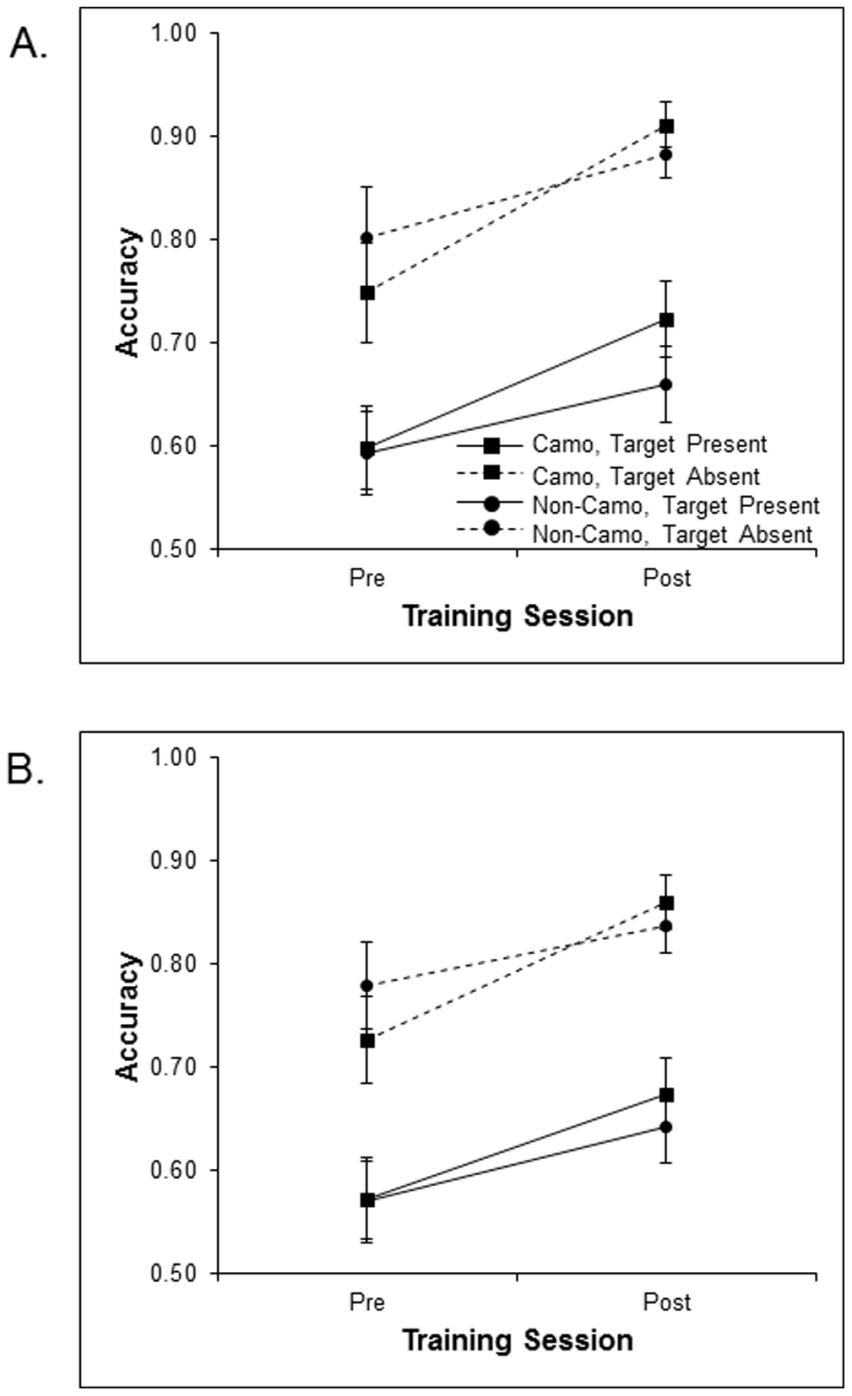
Mean accuracy across pre and post –training on untrained camouflage test displays for the camouflage and non-camouflage training groups as a function of target presence at set sizes 3 (A) and 5 (B).

A similar ANOVA was conducted on the camouflage background test data. The data are illustrated in Figure 6. There were main effects of target presence [*F* (1, 46) = 33.80, *p*<.001], set size [*F* (1, 46) = 14.52, *p*<.001], and session [*F* (1, 46) = 51.80, *p*<.001]. There was no main effect of training group [*F* (1, 46) = .06, *p* = .81, p_BIC_(H_0_|D) = .87], but importantly there was a significant interaction between session and training group [*F* (1, 46) = 4.88, *p*<.05]. Although both training groups showed improved accuracy in search for camouflaged targets at post-test, participants who were trained to search for camouflaged targets displayed larger accuracy improvements when searching for untrained camouflaged targets than participants who were not trained to search for camouflaged targets. This differential improvement at transfer suggests that camouflage training impacted the broader processes underlying search in general, rather than just a stimulus specific factor. To confirm that training benefits were related to changes in detection sensitivity, as opposed to changes in response biases, we also submitted data to signal detection analysis. When searching for camouflaged targets at post-test, camouflage trained participants exhibited higher sensitivity (d’ = 1.91) than non-camouflage trained participants (d’ = 1.62), despite having slightly lower target sensitivity at pre-test (d’ = 1.05 and 1.27 for camouflage and non-camouflage trained participants, respectively; patterns were similar across set sizes and thus data were collapsed across the factor). Critically, at post-test, both groups displayed similar response criterions (β = .64 and.67 for camouflage and non-camouflage trained participants, respectively), indicating that training resulted in actual search improvements and transfer benefits in camouflaged target-detection for the camouflage-training group compared to non-camouflage trained participants.

### Transfer Effects: Training Session 5 vs. Post-training Test

**Figure 7:**
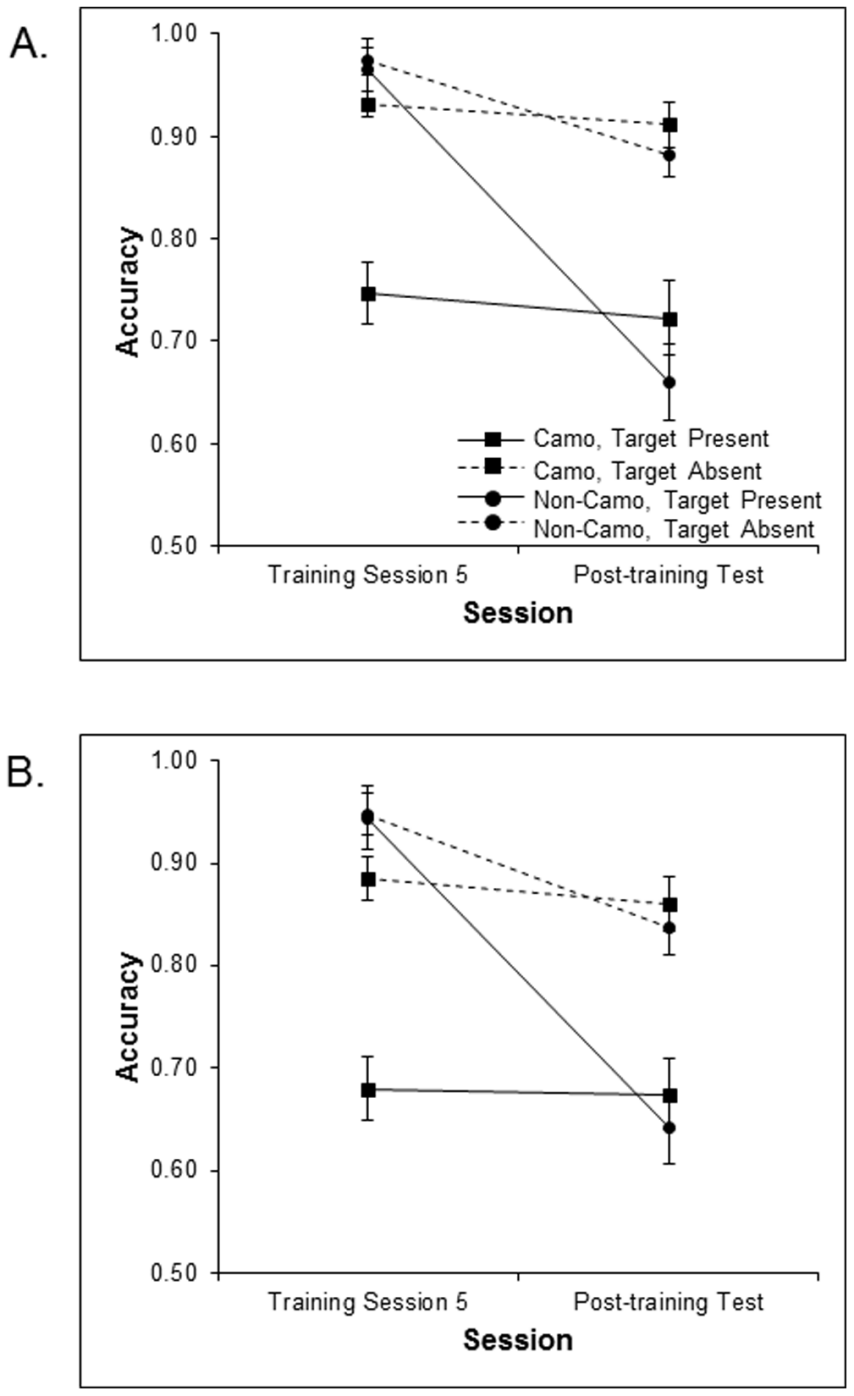
Mean accuracy across training session 5 and post –training on untrained camouflage test displays for the camouflage and non-camouflage training groups as a function of target presence at set sizes 3 (A) and 5 (B).

To further quantify whether training transferred to search performance for untrained camouflaged stimuli an ANOVA was conducted to compare performance on the post-training camouflage background test to that of the final training session (training session 5) (see Figure 7). Target presence (present vs. absent), set size (3 vs. 5), and session (training session 5 vs. post-training) were entered in an ANOVA as within-participants factors and training group (camouflage vs. non-camouflage) was entered as a between-participants factor. Of particular interest is the comparison between Training Session 5 and post-training camouflage test for the camouflage training group; a non-significant difference in this comparison would represent evidence for transfer of training. We found main effects of target presence [*F* (1, 46) = 46.51, *p*<.001], set size [*F* (1, 46) = 40.97, *p*<.001], training group [*F* (1, 46) = 5.20, *p*<.001], and session [*F* (1, 46) = 79.48, *p*<.001]. The main effects of training group and session appear to have been driven mainly by the non-camouflage training group, as reflected by a significant session by training group interaction [*F* (1, 46) = 55.08, *p*<.001]. To further characterize this interaction we ran ANOVA on both training groups separately. Of critical interest was whether a main effect of session, which would indicate a change in performance from training session 5 to post camouflage-test, was present for either group. As reflected by a significant main effect of session, participants who did not train on camouflage backgrounds demonstrated a large decline in accuracy when comparing performance on their final training session to that on the camouflage search post-test [*F* (1, 23) = 114.13, *p*<.001]. In contrast, and most importantly, participants who trained on camouflage search displayed near-perfect transfer of training at post-test; accuracy did not significantly decrease from training session 5 to the post-training camouflage test [*F* (1, 23) = 1.34, *p* = .26, p_BIC_(H_0_|D) = .72]. Though transfer of training is typically rare in the literature on perceptual learning, our results are consistent with previous studies of camouflage training that also found near perfect transfer of training [1], [4] and with findings of training-induced improvements in contrast sensitivity [11].

## Discussion

The purpose of this study was to investigate the efficacy of training and transfer of acquired skill in visual search for camouflaged targets. Whereas previous studies have explored this topic using participant-terminated search displays, we utilized a rapid display paradigm (150 ms) that minimized display induced eye movements. Our use of rapidly presented stimuli allowed us to disambiguate whether previously observed training and transfer effects were related to oculomotor strategy shifts (e.g., avoiding eye movements to salient objects that cannot be the target in a camouflage search task), covert attention shifts, or training induced changes in lower level perceptual processing.

Overall, we found that the camouflage training group showed accuracy improvements over training, a larger improvement in search accuracy during search of untrained camouflage displays compared to the non-camouflage trained group (although it should be noted that the non-camouflage training group showed some improvement on camouflage search displays from pre to post as well), and near perfect transfer of training from trained to similar, but untrained camouflage search displays. Our findings are broadly consistent with previous studies that found training related improvement in search for camouflaged targets and near perfect transfer of training to novel display [1], [4]. However, our study also suggests that these training improvements need not be associated with overt attention, as overt attention shifts in our paradigm were rare and omitted from analysis when they did occur. If training-related improvements in camouflaged target detection are not associated with higher-order strategy shifts in oculomotor attention then where might they arise from?

One possibility is that covert attention might be better guided, or more efficiently deployed (perhaps through the adoption of some standard spatial template) with training. This possibility is not directly testable given the current data, however, at least two points argue against such an arrangement underlying training improvement in our task. A first argument against covert attention underlying training and transfer improvements can be gleaned from previous studies in which training and transfer effects were found during search for camouflaged targets [1], [4]. Specifically, previous studies have employed search displays with off-set grid-like arrays of objects on target-similar backgrounds with higher set sizes (up to 19 objects per display). Although it is fairly easy to posit an attentional template that would improve search performance in the current paradigm, it is much harder to do so for previous paradigms in which the search arrays were larger, less predictable, and less structured. A second argument against an explanation of our results based solely on covert attention comes from previous studies showing that covert and overt attention are tightly coupled [12], [13]. Given the similarity of our findings using rapid presentation to those using free viewing paradigms in which eye movements were possible, it seems unlikely (though we cannot completely rule the possibility out) that covert attention alone can be implicated as underlying the converging observations of training and transfer.

Another and perhaps more likely candidate process underlying our training and transfer improvement is figure-ground texture segmentation. It has long been suggested that a key factor in breaking camouflage is figure-ground assignment; when the target can be segmented from the background, camouflage is more likely to be broken [14]. Recently, figure-ground segmentation has been shown to be amenable to training. Specifically, Yi and colleagues found that over the course of 16 training sessions participants became better at distinguishing symmetric dot patterns from backgrounds of random dot arrays [9]. Interestingly, they found that training on dot patterns arrayed on empty backgrounds did not engender better segmentation of dot patterns on random dot backgrounds. This pattern is consistent with our observation of improved performance on novel camouflage displays by our camouflage trained participants, but not by our non-camouflage trained participants. Chen and Hedge recently suggested that training-induced improvements in figure ground segmentation in camouflage search tasks might be accomplished through a mechanism by which observers learn the statistical properties of a background image [15]. To make figure-ground assignments observers need only compare the statistical properties of the background with the target present to those when the target is not. Our findings are not incompatible with this account, although it is interesting to note that in our study camouflage trained participants exhibited near perfect transfer to novel objects and backgrounds. This might suggest that is not the specific background that is important for learning, but rather the statistical properties associated with the broader structure of the background itself. Although the backgrounds tested at post-test in our study were indeed untrained, the same process was used to create those backgrounds as those that were trained. Hence, the general structure (a tile-like arrangement) of the untrained backgrounds was, to some degree, similar to those that were used for training. Finally, it is worth noting that improvements in feature-ground segmentation do not altogether preclude changes in covert attentional orienting. Instead, improved figure-ground segmentation arising from training might in turn increase the likelihood of accurate covert attentional shifts, providing a mechanism by which improvements in camouflaged target detection might be associated with a broader improvement in a network of related perceptual processes rather than one discrete mechanism. Future work will examine these possibilities in more detail.

## References

[1] Boot WR, Neider MB, Kramer AF (2009) Training and transfer in search for camouflaged real-world targets. Atten Percept Psychophys 71: 950–963. 19429971 10.3758/APP.71.4.950

[2] Wolfe JM, Oliva A, Horowitz TS, Butcher SJ, Bompas A (2002) Segmentation of objects from backgrounds in visual search tasks. Vision Res 42: 2985–3004. 12480070 10.1016/s0042-6989(02)00388-7

[3] Neider MB, Zelinsky GJ (2006) Searching for camouflaged targets: Effects of target–background similarity on visual search. Vision Res 46: 2217–2235. 16497351 10.1016/j.visres.2006.01.006

[4] Neider MB, Boot WR, Kramer AF (2010) Visual search for real world targets under conditions of high target-background similarity: Exploring training and transfer of training in older adults. Acta Psychol 134: 29–39. 10.1016/j.actpsy.2009.12.001 20038458

[5] Fiorentini A, Berardi N (1981) Learning in grating waveform discrimination: Specificity for orientation and spatial frequency. Vision Res 21: 1149–1158. 7314493 10.1016/0042-6989(81)90017-1

[6] Ahissar M, Hochstein S (1996) Learning pop-out detection: Specificities to stimulus characteristics. Vision Res 36: 3487–3500. 8977015 10.1016/0042-6989(96)00036-3

[7] Ellison A, Walsh V (1998) Perceptual learning in visual search: Some evidence of specificities. Vision Res 38: 333–345. 9536359 10.1016/s0042-6989(97)00195-8

[8] Stevens M, Merilaita S (2009) Animal camouflage: Current issues and new perspectives. Philos Trans R Soc Lond B Biol Sc 364: 423–427. 18990674 10.1098/rstb.2008.0217 PMC2674078

[9] Yi DJ, Olson IR, Chun MM (2006) Shape-specific perceptual learning in a figure-ground segregation task. Vision Res 46: 914–924. 16242752 10.1016/j.visres.2005.09.009

[10] Masson MEJ (2011) A tutorial on a practical Bayesian alternative to null-hypothesis significance testing. Behav Res Methods 43: 679–690. 21302025 10.3758/s13428-010-0049-5

[11] Li R, Polat U, Makous W, Bavelier D (2009) Enhancing the contrast sensitivity function through action video game playing. Nat Neurosci 12: 549–551. 19330003 10.1038/nn.2296 PMC2921999

[12] Hoffman JE, Subramaniam B (1995) The role of visual attention in saccadic eye movements. Percept Psychophys 57: 787–795. 7651803 10.3758/bf03206794

[13] Peterson MS, Kramer AF, Irwin DE (2004) Covert shifts of attention precede involuntary eye movements. Percept Psychophys 66: 398–405. 15283065 10.3758/bf03194888

[14] Cott HB (1948) Camouflage. Advancement of Science 4: 300–309. 18858266

[15] Chen X, Hegdé J (2012) Learning to break camouflage by learning the background. Psychol Sci 23: 1395–1403. 23064405 10.1177/0956797612445315

